# Asymmetric and symmetric dimethylarginine in high altitude pulmonary hypertension (HAPH) and high altitude pulmonary edema (HAPE)

**DOI:** 10.3389/fphys.2023.1297636

**Published:** 2023-11-29

**Authors:** Juliane Hannemann, Julius Freytag, Lisa Maria Schiefer, Franziska Macholz, Mahdi Sareban, Lena Schmidt-Hutten, Heike Stang, Edzard Schwedhelm, Erik R. Swenson, Rainer Böger, Marc Moritz Berger

**Affiliations:** ^1^ Institute of Clinical Pharmacology and Toxicology, University Medical Center Hamburg-Eppendorf, Hamburg, Germany; ^2^ Institute DECIPHER, German-Chilean Institute for Research on Pulmonary Hypoxia and its Health Sequelae, Hamburg, Germany; ^3^ Department of Anesthesiology and Intensive Care Medicine, University Hospital Essen, University Duisburg-Essen, Essen, Germany; ^4^ Paracelsus Medical University Salzburg, Salzburg, Austria; ^5^ Department of Anesthesiology, Critical Care and Pain Medicine, Paracelsus Medical University, Salzburg, Austria; ^6^ University Institute of Sports Medicine, Prevention and Rehabilitation and Research Institute of Molecular Sports Medicine and Rehabilitation, Paracelsus Medical University, Salzburg, Austria; ^7^ Division of Pulmonary, Critical Care and Sleep Medicine, VA Puget Sound Health Care System, University of Washington, Seattle, WA, United States

**Keywords:** nitric oxide, NO, ADMA (asymmetric dimethyl arginine), hypoxia, high altitude, biomarker

## Abstract

**Introduction:** High altitude exposure may lead to high altitude pulmonary hypertension (HAPH) and high altitude pulmonary edema (HAPE). The pathophysiologic processes of both entities have been linked to decreased nitric oxide (NO) availability.

**Methods:** We studied the effect of acute high altitude exposure on the plasma concentrations of asymmetric (ADMA) and symmetric dimethylarginine (SDMA), L-arginine, L-ornithine, and L-citrulline in two independent studies. We further investigated whether these biomarkers involved in NO metabolism were related to HAPH and HAPE, respectively. Fifty (study A) and thirteen (study B) non-acclimatized lowlanders were exposed to 4,559 m for 44 and 67 h, respectively. In contrast to study A, the participants in study B were characterized by a history of at least one episode of HAPE. Arterial blood gases and biomarker concentrations in venous plasma were assessed at low altitude (baseline) and repeatedly at high altitude. HAPE was diagnosed by chest radiography, and HAPH by measuring right ventricular to atrial pressure gradient (RVPG) with transthoracic echocardiography. AMS was evaluated with the Lake Louise Score (LLS) and the AMS-C score.

**Results:** In both studies SDMA concentration significantly increased at high altitude. ADMA baseline concentrations were higher in individuals with HAPE susceptibility (study B) compared to those without (study A). However, upon high altitude exposure ADMA only increased in individuals without HAPE susceptibility, while there was no further increase in those with HAPE susceptibility. We observed an acute and transient decrease of L-ornithine and a more delayed but prolonged reduction of L-citrulline during high altitude exposure. In both studies SDMA positively correlated and L-ornithine negatively correlated with RVPG. ADMA was significantly associated with the occurrence of HAPE (study B). ADMA and SDMA were inversely correlated with alveolar PO_2_, while L-ornithine was inversely correlated with blood oxygenation and haemoglobin levels, respectively.

**Discussion:** In non-acclimatized individuals ADMA and SDMA, two biomarkers decreasing endothelial NO production, increased after acute exposure to 4,559 m. The observed biomarker changes suggest that both NO synthesis and arginase pathways are involved in the pathophysiology of HAPH and HAPE.

## Introduction

Acute exposure of non-acclimatized individuals to high altitude is associated with the risk of developing acute mountain sickness (AMS) and high altitude pulmonary edema (HAPE) which are the two most frequent forms of acute high altitude illnesses (Bärtsch and Swenson, 2013; Luks and Hackett, 2022). AMS is a complex of nonspecific symptoms and manifests itself as headache, fatigue, gastrointestinal discomfort, and dizziness occurring within the first days after ascent to altitude >2,500 m (Bärtsch et al., 2004; Roach et al., 2018). HAPE is a non-cardiogenic pulmonary edema that may develop within 1–5 days after acute exposure to altitudes >3,000 m (West, 2012). A main pathophysiological factor in the origin of HAPE is excessive hypoxic pulmonary vasoconstriction (HPV) (Dehnert et al., 2007) leading to elevation in pulmonary vascular resistance with time and high altitude pulmonary hypertension (HAPH) (Ghofrani et al., 2006; Swenson, 2013; Brito et al., 2018). Therefore, HAPH and HAPE are inter-related clinical conditions that may relate to similar or overlapping pathophysiological mechanisms.

The pathophysiology of AMS and HAPE is complex, and the specific molecular causes are not completely understood. While endothelial dysfunction with decreased nitric oxide (NO) availability is considered to play a major role in HAPE (Scherrer et al., 1996; Berger et al., 2005; Swenson and Bärtsch, 2012), it has been suggested that increasing plasma concentrations of nitrate, an NO metabolite, may exacerbate AMS (Rossetti et al., 2017).

NO is the major endothelium-derived vasodilator; in the lungs, NO counterbalances vasoconstrictor stimuli in the physiological regulation of hypoxic pulmonary vasoconstriction (Swenson, 2013; Böger and Hannemann, 2020). The activity of endothelial NO synthase (eNOS), which generates NO and L-citrulline from L-arginine, is competitively inhibited by asymmetric dimethylarginine (ADMA), a dimethylated derivative of L-arginine (Böger, 2006). By contrast, symmetric dimethylarginine (SDMA) does not directly interfere with eNOS activity, but both ADMA and SDMA inhibit the cellular uptake of L-arginine and thereby impair eNOS substrate availability (Hannemann and Böger, 2022). As cellular uptake of L-arginine is inhibited by both dimethylarginines in a manner competitive with L-arginine, it is probable but has never been experimentally shown yet that both dimethylarginine act in an additive manner via this molecular mechanism. We have shown previously that prolonged exposure of humans to chronic intermittent hypobaric hypoxia (CIHH) causes elevation of ADMA plasma concentration (Lüneburg et al., 2016; Siques et al., 2019). Data on SDMA are less clear. Animal studies in rats and mice exposed to acute or chronic hypoxia have confirmed these observations and have shown that downregulation of dimethylarginine dimethylaminohydrolase-1 (DDAH1), the major metabolizing enzyme for ADMA, is involved in this ADMA increase (Hannemann et al., 2020a). Other investigators reported downregulation of DDAH2 in a rat model of CIHH (López et al., 2021); however, this data stands in contrast to our findings from a mouse model (Hannemann et al., 2020b) and those from a rat model (Lüneburg et al., 2016). Several studies have also provided evidence that arginase, an enzyme that uses L-arginine as a substrate to generate L-ornithine, may also be upregulated in chronic intermittent hypoxia (Krause et al., 2015) and in CIHH (López et al., 2021). Therefore, L-arginine-related biochemical pathways seem to be involved in the body’s response to high altitude hypoxia in a multi-facetted, complex way.

**FIGURE 1:**
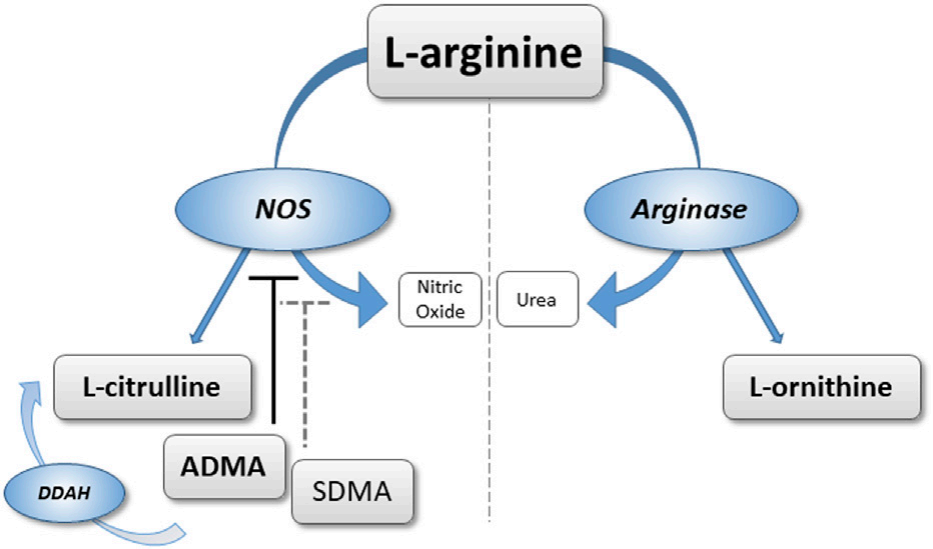
Schematic representation of the L-arginine/dimethylarginine/nitric oxide (NO) pathway. L-arginine can be converted to NO and L-citrulline via NO synthase, or to L-ornithine via arginase. Asymmetric dimethylarginine (ADMA) and symmetric dimethylarginine (SDMA) are released from methylated proteins. ADMA directly inhibits NO synthase; ADMA and SDMA interfere with cellular L-arginine uptake.

Previous data from our group have suggested that elevated ADMA is a pathophysiological contributor to HAPH and a biomarker characterizing individuals at high risk of developing HAPH during prolonged exposure to CIHH (Hannemann et al., 2020a). In two different cohorts of healthy male individuals who were exposed to four and 6 months of chronic intermittent hypoxia, respectively, we observed gradual increases in ADMA plasma concentration as early as at 1 month (Lüneburg et al., 2016; Siques et al., 2019). To assess whether high altitude hypoxia leads to acute increases of ADMA and SDMA in healthy humans, and to study whether these mediators are associated with HAPH and HAPE during short-term exposure to high altitude, we re-analyzed plasma samples taken in two separate cohorts exposed to an altitude of 4,559 m. Additional metabolites were also measured that are products of NOS- (L-citrulline) and arginase- (L-ornithine) mediated metabolism of L-arginine. Figure 1 schematically displays the L-arginine–dimethylarginine–nitric oxide pathway to allow for clarification of the biochemical pathways represented by each metabolite.

## Methods

### Study participants and protocols

We analyzed plasma samples stored frozen at −80°C from two separate high-altitude studies. In both studies, healthy, non-acclimatized individuals ascended within 20 h from 1,130 to 4,559 m (Capanna Regina Margherita), with an intervening overnight stay at 3,611 m (Gnifetti Hut). The designs and main outcomes of both studies have been published previously (Berger et al., 2018; Berger et al., 2022).

In study A, conducted in 2016, 50 individuals were randomly and prospectively assigned to inhaled budesonide (200 or 800 µg twice per day) or placebo, and assessed for 44 h at high altitude (Berger et al., 2017). Treatment started 1 day prior to ascent and continued until the end of the study. All measurements were done at low altitude (baseline, 423 m) and 7, 20, 32, and 44 h after ascent to high altitude.

In study B, conducted in 2019, thirteen lowlanders with a previous history of radiographically documented HAPE were randomly assigned to acetazolamide (250 mg orally three times per day) or placebo. Medication started 2 days before ascent and continued until the end of the study (Berger et al., 2022). Measurements were done at low altitude (baseline, 423 m) and 5, 19, 29, 43, 53, and 67 h after ascent to high altitude. Both studies were approved by the Ethics Committee of Salzburg, Austria (study A: 415-E/1998/6-2016; study B: 415-E/2290/7-2018). The studies had also been approved by the Ethical Committee of the University of Torino, Italy (study A: 44847 del 13/5/2016; study B: 435581 del 6/11/2018), and by the Austrian Competent Authority (BASG), Vienna, Austria (study A: 8968266; study B: 10578359). All participants provided written informed consent before inclusion into the studies.

Because in both studies the study medication had no significant effect on the plasma concentrations of the biomarkers of interest for this study, the data of all individuals in study A, and of all individuals in study B, respectively, were pooled for final analyses.

### Assessment of right ventricular pressure gradient, high altitude pulmonary edema, and acute mountain sickness

As described previously in detail (Berger et al., 2017; Berger et al., 2022) transthoracic echocardiography was performed to assess the right ventricular to atrial pressure gradient as a surrogate parameter for pulmonary artery pressure, and chest radiography for diagnosing HAPE. HAPH was defined as systolic pulmonary artery pressure >50 mmHg according to the definition by (León-Velarde et al., 2005).

AMS was evaluated by the AMS-C score of the abbreviated version of the Environmental Symptoms Questionnaire (Beidleman et al., 2007). Individuals were considered AMS-positive when they had an AMS-C score ≥0.70 points (Berger et al., 2017). In addition, the Lake Louise score (LLS) was assessed; in study A with the original (Roach, 1993), and in study B with its revised version (Roach et al., 2018).

### Blood drawings and additional measurements

Blood gas analyses were performed on capillary blood in study A (Siemens, RapidPoint 500, Germany), and on arterial blood samples collected from the radial artery in study B (safePICO, Radiometer, Brønshøj, Denmark). Serial venous blood samples were drawn from a cubital vein, and aliquots of samples were shock frozen in liquid nitrogen until stored at −80°C. Oxygen saturation (SpO_2_) was measured by pulse oximetry (Covidien Nellcor, Mansfield, United States). In study A the alveolar P_O2_ was calculated from the alveolar gas equation (Fenn et al., 1946) assuming a respiratory exchange ratio of 0.85 (West et al., 1983) and an alveolar P_CO2_ equal to capillary P_CO2_. These assessments were not performed in study B.

### Measurement of plasma concentrations of dimethylarginines, L-arginine, L-citrulline, and L-ornithine

Validated protocols for ultra-performance liquid chromatography-tandem mass spectrometry (UPLC-MS/MS) were used to quantify ADMA, SDMA, L-arginine, L-citrulline, and L-ornithine in plasma. Briefly, 25 μL of plasma were diluted in 100 µL methanol to which stable isotope labelled internal standards had been added. Subsequently, the compounds were converted into their butyl ester derivatives as described elsewhere (Schwedhelm et al., 2007). Quantification of analytes was performed on a Waters UPLC-MS/MS platform (Xevo TQ-S cronos, Waters GmbH, Eschborn, Germany) applying an ACQUITY UPLC BEH C_18_ column (2.1 × 50 mm, 1.7 μm, Waters GmbH) for chromatographic separation. The coefficient of variation for the quality control samples was below 6% for all compounds.

### Statistical analyses

Normal distribution of the data was tested using the Kolmogorov–Smirnov test. Differences in biomarker concentrations over time within study A and study B, respectively, were analyzed by two-way repeated-measures ANOVA with group and time as variables, and using the Bonferroni correction for multiple testing. Pairwise multiple-comparison procedures were made by using the Student–Newman–Keuls test. The relationship between pairs of variables was expressed with the Pearson Correlation Coefficient (R). All data are presented as mean ± standard deviation. A *p* < 0.05 after adjustment for multiple testing was considered significant. Statistics were performed using the SigmaStat software packages (Systat Software Inc., Berkshire, United Kingdom).

## Results

### Baseline characteristics and biomarker concentrations

The baseline demographic and anthropometric data of the two study cohorts are shown in Table 1. In both studies, the biomarker plasma concentrations at baseline were within the respective reference ranges (Schwedhelm et al., 2009; Lüneburg et al., 2011; Schwedhelm et al., 2011; Table 2). In participants with HAPE susceptibility (study B), the plasma concentrations of ADMA (0.54 ± 0.06 vs. 0.41 ± 0.06 μmol/L; *p* < 0.001) and SDMA (0.53 ± 0.08 vs. 0.47 ± 0.07 μmol/L; *p* = 0.012) as well as those of L-arginine (76.4 ± 11.7 vs. 62.9 ± 13.1 μmol/L; *p* < 0.001) and L-citrulline (36.9 ± 7.4 vs. 30.5 ± 5.6 μmol/L; *p* = 0.001) were significantly higher than in individuals without HAPE susceptibility (study A). The difference in L-ornithine plasma concentration did not reach statistical significance (*p* = 0.056).

**TABLE 1:** Baseline characteristics of the study cohorts.

	Non-HAPE-susceptible	HAPE-susceptible
Demographics		
No. of individuals	50	13
Age (years)	43 ± 11	57 ± 6
Sex (f/m)	16/34	2/11
Anthropometrics		
Height (cm)	176 ± 9	175 ± 7
Weight (kg)	72 ± 11	76 ± 11
Systolic blood pressure (mmHg)	123 ± 12	130 ± 19
Diastolic blood pressure (mmHg)	72 ± 7	85 ± 7
Heart rate (1/min)	61 ± 10	63 ± 10
RVPG at baseline* (mmHg)	19 ± 4	20 ± 5
RVPG at altitude* (mmHg)	34 ± 8	43 ± 10
Incidence of HAPH	10	8
Incidence of HAPE	0	7

Baseline measurements were performed at low altitude (423 m above sea level in Salzburg, Austria). Data are numbers of individuals for categorical variables and mean ± standard deviation for continuous variables. Abbreviation: RVPG, right ventricular pressure gradient. *RVPG, at baseline represents the measurement at low altitude; RVPG, at altitude represents the first measurement after ascent to high altitude (Study A: 7 h, study B: 5 h).

**TABLE 2:** Biomarker concentrations and biomarker ratios at baseline (low altitude) and at various time points after ascent to high altitude.

Non-HAPE-susceptible	Baseline	7 h	20 h	32 h	44 h			P_group_	P_time_	P_group x time_
ADMA	0.41 ± 0.06	0.42 ± 0.06	0.45 ± 0.08	0.45 ± 0.08	0.46 ± 0.05			0.31	<0.001	0.59
SDMA	0.47 ± 0.07	0.51 ± 0.07	0.53 ± 0.08	0.52 ± 0.08	0.51 ± 0.07			0.06	<0.001	0.55
L-Arginine	62.9 ± 13.1	59.0 ± 12.3	63.4 ± 14.0	62.3 ± 12.9	68.4 ± 11.7			0.36	<0.001	0.21
L-Ornithine	53.4 ± 12.7	44.9 ± 7.9	46.2 ± 8.9	47.8 ± 10.3	63.3 ± 9.5			0.95	<0.001	0.96
L-Citrulline	30.5 ± 27.3	27.3 ± 5.4	26.9 ± 5.1	25.9 ± 4.1	29.5 ± 4.4			0.79	<0.001	0.13
Arg/ADMA Ratio	153.6 ± 32.4	142.5 ± 29.3	143.7 ± 30.2	140.5 ± 29.5	150.0 ± 27.2			0.39	<0.001	0.16
Cit/Arg Ratio	0.50 ± 0.12	0.48 ± 0.11	0.44 ± 0.10	0.43 ± 0.10	0.44 ± 0.08			0.68	<0.001	0.90
Orn/Arg Ratio	0.87 ± 0.22	0.79 ± 0.18	0.76 ± 0.18	0.80 ± 0.25	0.80 ± 0.18			0.44	<0.001	0.50

HAPE-susceptible	Baseline	5 h	19 h	29 h	43 h	53 h	67 h	P_group_	P_time_	P_group x time_
ADMA	0.54 ± 0.06	0.52 ± 0.05	0.51 ± 0.05	0.53 ± 0.05	0.53 ± 0.05	0.53 ± 0.06	0.56 ± 0.08	0.39	0.01	0.77
SDMA	0.53 ± 0.08	0.58 ± 0.09	0.58 ± 0.09	0.58 ± 0.10	0.58 ± 0.06	0.57 ± 0.07	0.54 ± 0.08	0.39	0.02	0.69
L-Arginine	76.4 ± 11.7	69.2 ± 8.8	68.5 ± 10.6	70.8 ± 10.6	72.9 ± 8.7	70.4 ± 5.6	77.4 ± 12.3	0.62	0.005	0.98
L-Ornithine	60.9 ± 10.3	47.5 ± 8.0	45.2 ± 7.3	48.5 ± 9.4	49.6 ± 9.6	48.4 ± 9.4	55.8 ± 7.2	0.70	<0.001	0.95
L-Citrulline	36.9 ± 7.4	34.3 ± 8.5	33.5 ± 7.3	30.7 ± 5.9	28.0 ± 6.7	27.2 ± 5.5	31.6 ± 3.9	0.42	<0.001	0.94
Arg/ADMA Ratio	141.6 ± 22.1	136.3 ± 27.8	135.8 ± 28.4	135.7 ± 23.7	139.1 ± 24.1	134.7 ± 21.8	138.2 ± 20.2	0.80	0.34	0.37
Cit/Arg Ratio	0.49 ± 0.10	0.50 ± 0.11	0.50 ± 0.09	0.44 ± 0.06	0.39 ± 0.07	0.39 ± 0.06	0.41 ± 0.03	0.19	<0.001	0.96
Orn/Arg Ratio	0.80 ± 0.12	0.69 ± 0.11	0.67 ± 0.09	0.69 ± 0.10	0.68 ± 0.11	0.68 ± 0.09	0.73 ± 0.09	0.16	0.001	0.90

Biomarker concentrations are presented in µmol/L and show the mean ± standard deviation of all treatment groups combined within each study. *p* values are from two-way repeated measures ANOVA., P_group_ = difference between the different treatment groups (i.e., different concentrations of budesonide in study A, and of acetazolamide in study B); P_time_ = difference over time; P_group x time_ = degree of interaction between factor A (group) and B (time). Abbreviations: ADMA, asymmetric dimethylarginine; Cit/Arg ratio, L-citrulline/L-arginine ratio; Orn/Arg ratio, L-ornithine/L-arginine ratio; SDMA, symmetric dimethylarginine. Bold values are the significant *p* values.

### Effects of high altitude exposure on biomarker concentrations

**FIGURE 2:**
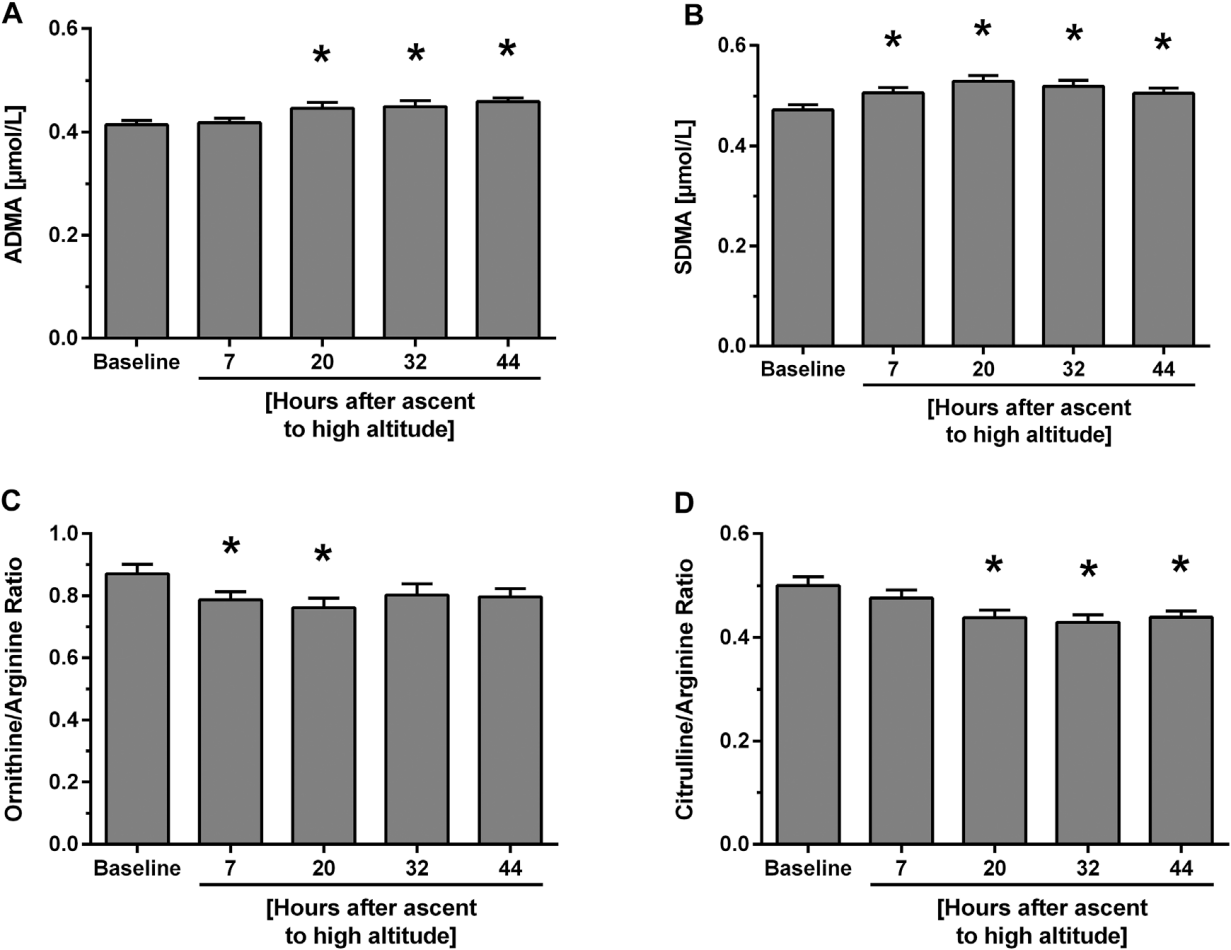
Time course of biomarker plasma concentrations and ratios at baseline and after ascent to high altitude (Study A). Data show concentrations of ADMA **(A)** and SDMA **(B)** as well as L-ornithine/L-arginine ratio **(C)** and L-citrulline/L-arginine ratio **(D)**. *p < 0.05 vs. baseline.

***Study A***. In individuals without known HAPE susceptibility, acute exposure to 4,559 m caused a mild, but significant and incremental increase in ADMA plasma concentration, which was significant for measurements taken between 20 and 44 h of altitude exposure (Figure 2A). SDMA also increased significantly with a peak at 20 h after ascent (Figure 2B). Plasma concentrations of L-arginine showed no consistent pattern over time at high altitude, whilst those of L-citrulline and L-ornithine decreased rapidly after ascent and returned to baseline levels at 44 h after ascent. As a consequence, L-arginine/ADMA ratio and L-ornithine/L-arginine ratio (Figure 2C) showed a significant decline from 7 h of altitude exposure on with some recovery at 44 h, whilst L-citrulline/L-arginine ratio showed a delayed but persistent decrease from 20 h of altitude exposure on (Figure 2D). All biomarker concentrations at all time points are given in Table 2.

**FIGURE 3:**
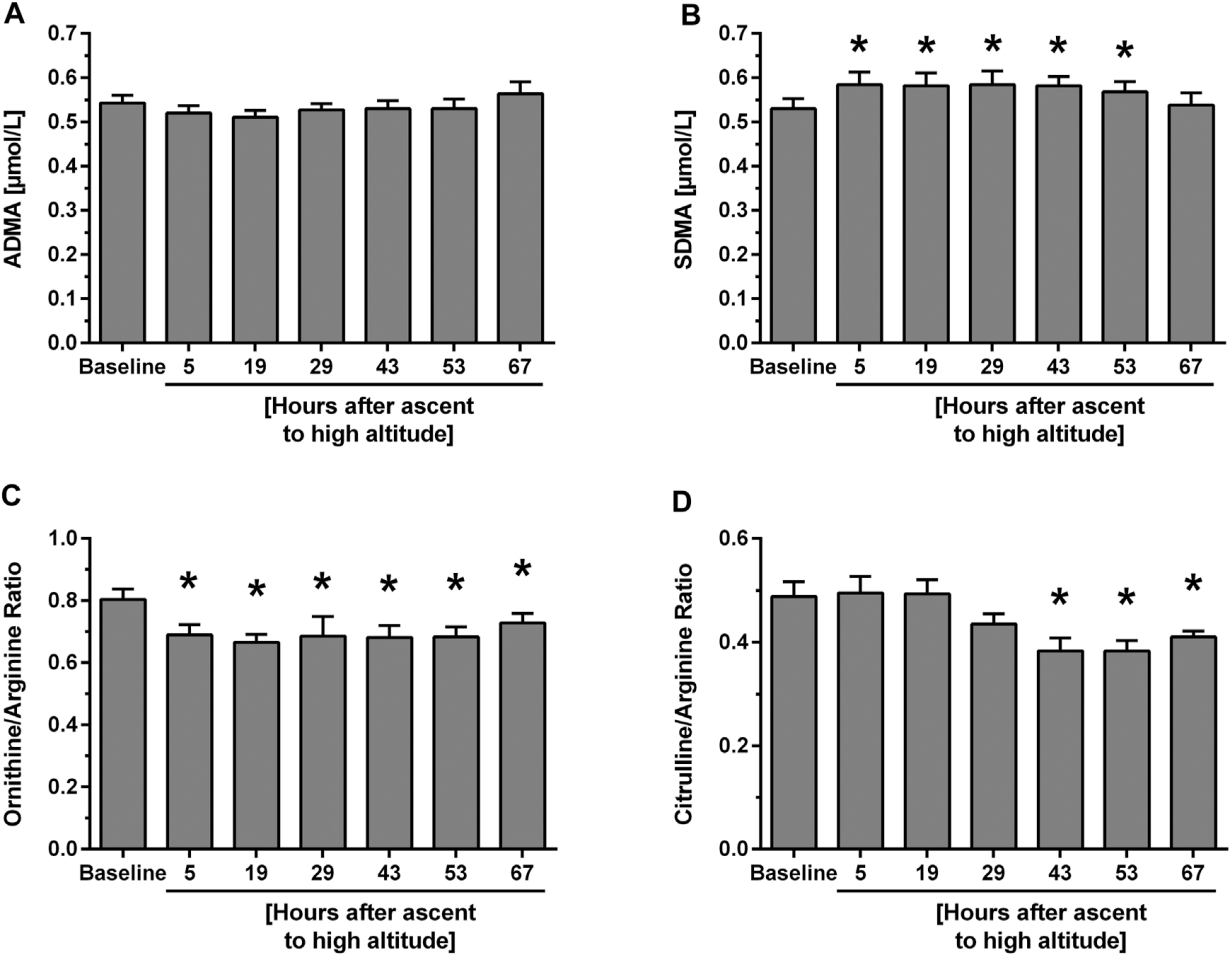
Time course of biomarker plasma concentrations and ratios at baseline and after ascent to high altitude (Study B). Data show concentrations of ADMA **(A)** and SDMA **(B)** as well as L-ornithine/L-arginine ratio **(C)** and citrulline/L-arginine ratio **(D)**. *p < 0.05 vs. baseline.

***Study B***. In participants with HAPE susceptibility ADMA baseline levels were higher than in those without HAPE susceptibility. However, there was no significant further elevation of ADMA during the 67 h of altitude exposure (Figure 3A). By contrast, SDMA again showed an initial, significant increase which peaked at 29 h and returned to baseline levels thereafter (Figure 3B). L-Arginine showed no consistent pattern of change with time at high altitude, whilst L-ornithine significantly dropped initially and returned to baseline at 67 h. L-citrulline showed a delayed decrease beyond 29 h of altitude exposure (Table 2). In this cohort, L-arginine/ADMA ratio remained unchanged during altitude exposure, whilst L-ornithine/L-arginine ratio acutely dropped after ascent to altitude (Figure 3C) and L-citrulline/L-arginine ratio was significantly reduced beyond 43 h of altitude exposure (Figure 3D).

In neither of the two studies did we detect any significant effect of the respective pharmacologic treatments (i.e., budesonide and acetazolamide, respectively) on biomarker levels as compared to the control groups (indicated by “P_group_” in Table 2), nor were there any significant effects of the pharmacological treatments on clinical diagnosis of HAPH or HAPE, as reported before (Berger et al., 2018; Berger et al., 2022). Therefore, all biomarkers were analysed for the complete study cohort without differentiation of treatment groups.

### Associations of biomarkers with high altitude right ventricular pressure gradient, high altitude pulmonary edema, and acute mountain sickness

**FIGURE 4:**
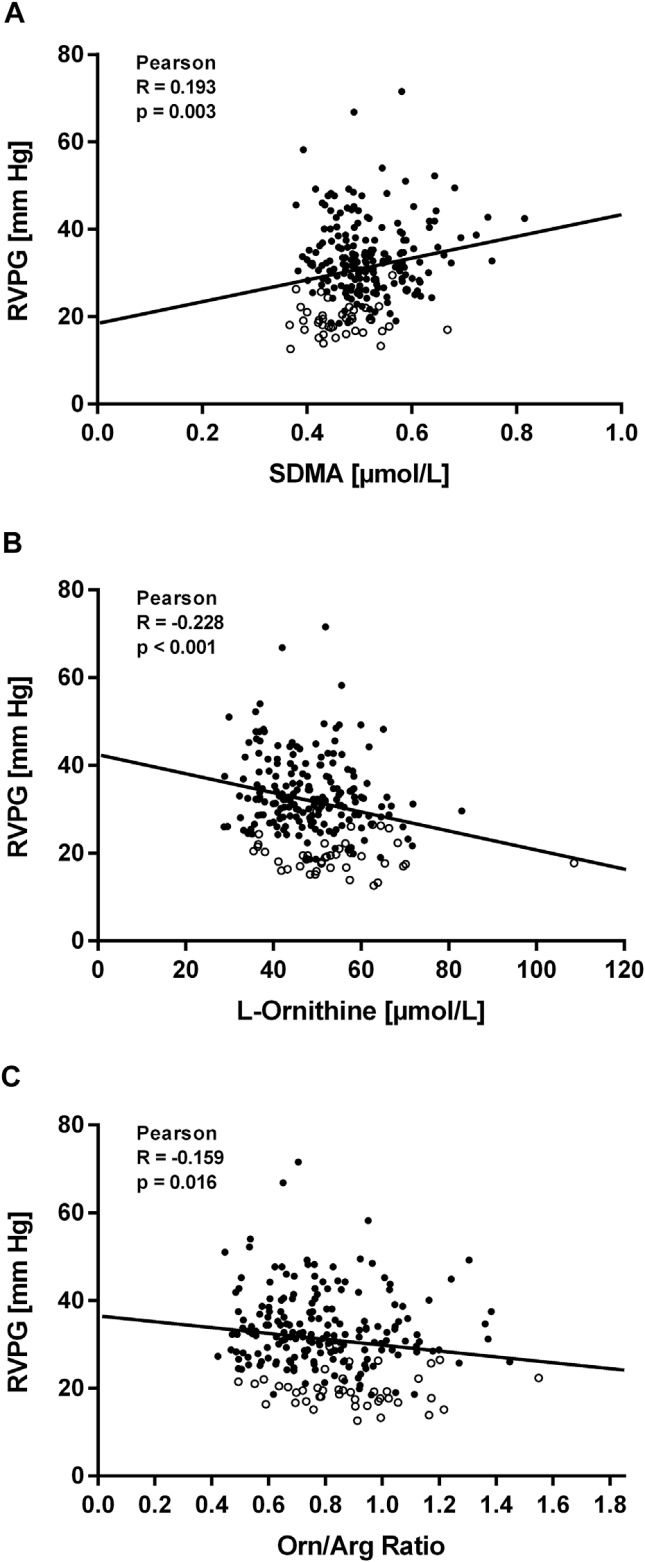
Correlations of biomarkers with right ventricular pressure gradient (RVPG) in participants of study **(A)**. Data are shown for SDMA **(A)**, L-ornithine **(B)**, and L-ornithine/L-arginine ratio **(C)**. Open symbols represent baseline measurements taken at low altitude, filled symbols represent measurements taken at high altitude at the times specified in Methods.

**FIGURE 5:**
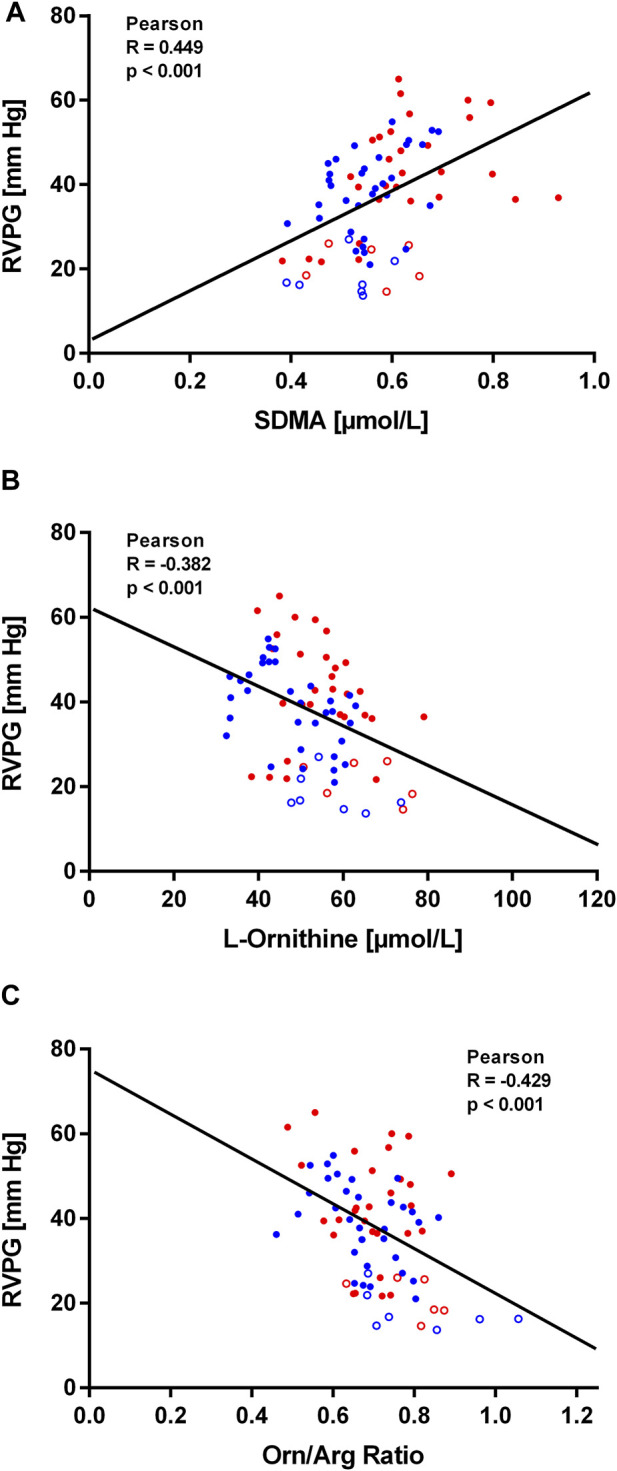
Correlations of biomarkers with right ventricular pressure gradient (RVPG) in participants of study **(B)**. Data are shown for SDMA **(A)**, L-ornithine **(B)**, and L-ornithine/L-arginine ratio **(C)**. Red symbols signify data from individuals who developed HAPE, blue symbols signify data from individuals who did not develop HAPE. Open symbols represent baseline measurements taken at low altitude, filled symbols represent measurements taken at high altitude at the times specified in Methods.

Within the first hours after ascent to high altitude RVPG increased to 34 ± 8 mmHg in those without HAPE susceptibility (study A) and to 43 ± 10 mmHg in those with HAPE susceptibility (study B). We observed a significant linear correlation of SDMA plasma concentration with RVPG in study A (Figure 4A) as well as in study B (Figure 5A). In neither of the two studies was there a significant correlation of ADMA with RVPG (not shown). Further, L-ornithine plasma concentration was significantly and inversely associated with RVPG in individuals without (Figure 4B) and with HAPE susceptibility (Figure 5B), as was the L-ornithine/L-arginine ratio (Figures 4C, 5C). All other biomarkers showed weak or no correlations with RVPG (Table 3).

**TABLE 3:** Correlations of biomarkers with right ventricular pressure gradient.

Biomarker	Non-HAPE-susceptible (study A)		HAPE-susceptbile (study B)	
	Pearson R	P	Pearson R	p
ADMA	0.060	0.327	−0.230	**0.047**
SDMA	0.193	**0.003**	0.449	**< 0.001**
L-Arginine	−0.010	0.835	−0.070	0.546
L-Citrulline	−0.070	0.253	−0.040	0.710
L-Ornithine	−0.228	**< 0.001**	−0.382	**< 0.001**
Arg/ADMA Ratio	−0.070	0.305	0.009	0.453
Cit/Arg Ratio	−0.070	0.320	−0.014	0.902
Orn/Arg Ratio	−0.159	**0.016**	−0.429	**< 0.001**

Abbreviations: ADMA, asymmetric dimethylarginine; Cit/Arg ratio, L-citrulline/L-arginine ratio; Orn/Arg ratio, L-ornithine/L-arginine ratio; SDMA, symmetric dimethylarginine. Bold values are the significant *p* values.

**FIGURE 6:**
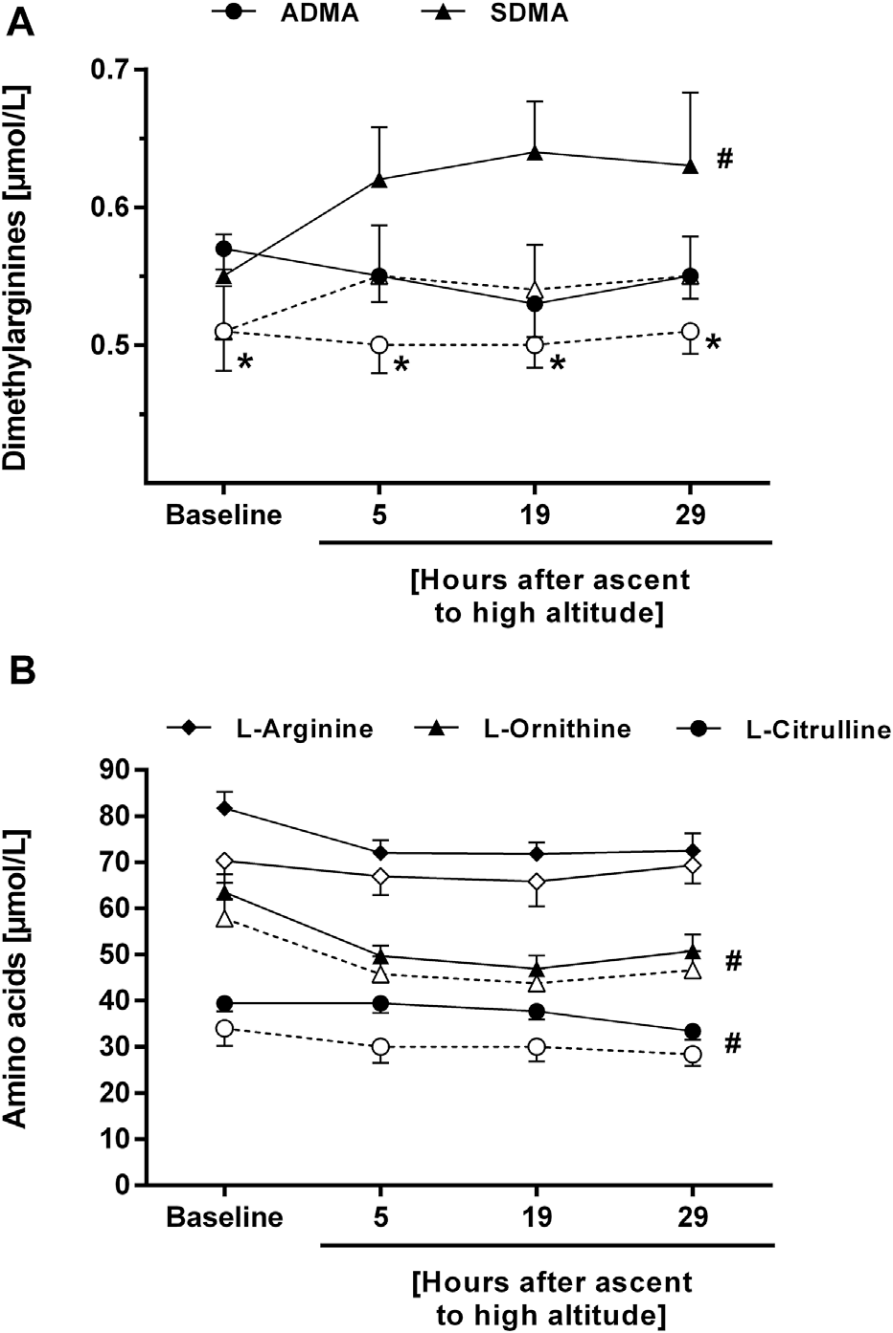
Time course of the plasma concentrations of ADMA and SDMA **(A)**, and of L-arginine, L-ornithine, and L-citrulline **(B)** in participants of study B who developed HAPE (filled symbols and solid lines) or not (open symbols and dashed lines). *p < 0.05 vs. participants without HAPE at the same time point; #*p* < 0.05 for time course of biomarker during altitude exposure vs. baseline.

In study B, seven out of thirteen study participants developed HAPE within 29 h after ascent to high altitude. Individuals who developed HAPE had a significantly higher mean baseline ADMA concentration than those who did not (0.57 ± 0.04 vs. 0.51 ± 0.07 μmol/L, *p* = 0.04). The difference in ADMA between both subgroups was maintained during the stay at high altitude, whilst SDMA showed a significant increase over time in both subgroups (Figure 6A). The concentrations of L-citrulline and L-ornithine significantly decreased with time of high altitude exposure, whilst there was no significant change in L-arginine concentration (Figure 6B).

In study A, L-citrulline and L-ornithine levels were negatively correlated, and SDMA concentration was positively correlated with AMS severity as reflected by both the AMS-C score and the LLS (Table 4). In study B, the negative correlation of L-ornithine with AMS severity was confirmed (Table 5), while none of the other biomarkers was associated with AMS severity. In none of the two studies there was a significant difference in any of the biomarkers between those having and not having AMS.

**TABLE 4:** Correlations of biomarkers with physiological responses to hypoxia and acute mountain sickness in individuals without HAPE susceptibility (study A).

Biomarker	Hb	PO_2alv_	AaDO_2_	SpO_2_	AMS-C	LLS
ADMA	−0.05/0.471	**−0.158/0.015**	**−0.171/0.008**	−0.050/0.427	0.016/0.811	0.077/0.235
SDMA	0.026/0.694	**−0.233/<0.001**	−0.121/0.060	**−0.29/<0.001**	**0.131/0.044**	**0.252/<0.001**
L-Arginine	−0.060/0.326	−0.013/0.841	**−0.149/0.02**	**0.129/0.046**	−0.060/0.371	−0.070/0.264
L-Citrulline	−0.010/0.837	**0.202/0.002**	0.002/0.719	**0.199/0.002**	**−0.167/0.010**	**−0.185/0.004**
L-Ornithine	**0.201/0.002**	**0.192/0.003**	−0.025/0.698	**0.292/<0.001**	**−0.246/<0.001**	**−0.332/<0.001**
Arg/ADMA Ratio	−0.006/0.929	0.110/0.090	−0.040/0.537	**0.180/0.005**	−0.101/0.119	**−0.157/0.015**
Cit/Arg Ratio	0.030/0.645	**0.183/0.005**	**0.162/0.012**	0.070/0.264	−0.081/0.212	−0.098/0.132
Orn/Arg Ratio	**0.207/0.001**	**0.150/0.021**	0.086/0.187	**0.129/0.047**	**−0.131/0.044**	**−0.190/0.003**

Values shown are Pearson correlation coefficients/*p* values. *values for AMS, indicate statistical significance comparing plasma concentrations of the respective biomarkers in study participants with and without acute mountain sickness. Abbreviations: AaDO_2_, alveolar-to-arterial difference in partial oxygen pressure ADMA, asymmetric dimethylarginine; AMS, acute mountain sickness; AMS-C, acute mountain sickness-cerebral score; Cit/Arg ratio, L-citrulline/L-arginine ratio; Hb, blood hemoglobin concentration; LLS, lake louise score; Orn/Arg ratio, L-ornithine/L-arginine ratio; PO_2alv_, partial oxygen pressure in alveolar air; SDMA, symmetric dimethylarginine; SpO_2_, blood oxygen saturation. Bold values are the significant *p* values.

**TABLE 5:** Correlations of biomarkers with physiological responses to hypoxia and acute mountain sickness in individuals with HAPE susceptibility (study B).

Biomarker	Hb	SaO_2_	PO_2art_	SpO_2_	AMS-C	LLS
ADMA	0.065/0.673	0.040/0.794	0.036/0.817	−0.012/0.924	−0.167/0.168	−0.195/0.109
SDMA	0.085/0.578	−0.132/0.387	0.055/0.718	−0.198/0.105	−0.074/0.545	−0.009/0.936
L-Arginine	−0.249/0.990	0.228/0.133	0.080/0.601	**0.288/0.017**	−0.086/0.477	−0.236/0.050
L-Citrulline	**−0.334/0.025**	0.271/0.072	**0.318/0.033**	0.122/0.323	0.045/0.712	−0.023/0.853
L-Ornithine	**−0.376/0.011**	**0.525/<0.001**	**0.377/0.011**	**0.483/<0.001**	−0.213/0.070	**−0.329/0.006**
Arg/ADMA Ratio	**−0.302/0.044**	0.183/0.229	**0.486/<0.001**	0.219/0.072	0.070/0.566	−0.035/0.774
Cit/Arg Ratio	−0.149/0.328	0.086/0.574	**0.436/0.003**	−0.064/0.605	0.115/0.344	0.132/0.281
Orn/Arg Ratio	−0.199/0.190	**0.449/0.002**	−0.172/0.258	**0.368/0.002**	−0.193/0.110	−0.210/0.083

Values shown are Pearson correlation coefficients/*p* values. *values for AMS, indicate statistical significance comparing plasma concentrations of the respective biomarkers in study participants with and without acute mountain sickness. Abbreviations: ADMA, asymmetric dimethylarginine; AMS, acute mountain sickness; AMS-C, acute mountain sickness-cerebral score; Cit/Arg ratio, L-citrulline/L-arginine ratio; Hb, blood hemoglobin concentration; LLS, lake louise score; Orn/Arg ratio, L-ornithine/L-arginine ratio; PO_2art_, partial oxygen pressure in arterial blood; SDMA, symmetric dimethylarginine; SaO_2_, arterial oxygen saturation in arterial blood gas analysis; SpO_2_, blood oxygen saturation in pulse oxymetry. Bold values are the significant *p* values.

### Associations of biomarkers with parameters of blood gas analyses

In study A, ADMA and SDMA were inversely correlated with alveolar PO_2_, and ADMA was also inversely correlated with the alveolar-arterial O_2_ difference. L-citrulline and L-ornithine both correlated positively with alveolar PO_2_, and L-ornithine also correlated positively with SpO_2_ (Table 4). In study B, the positive correlation of L-ornithine with SpO_2_ was confirmed, and both L-citrulline and L-ornithine also showed positive correlations with arterial PO_2_ (Table 5). We observed a significant correlation of L-ornithine concentration with hemoglobin levels (R = 0.201, *p* = 0.002) in study A. None of the other biomarkers was significantly correlated with hemoglobin. In study B, L-ornithine showed an inverse correlation with hemoglobin.

## Discussion

The key finding of our study is that the concentrations of ADMA and SDMA in plasma increase significantly within 24 h after ascent to high altitude. Concomitantly with this increase in dimethylarginine concentrations, the plasma levels of L-citrulline and L-ornithine showed a significant, transient decline, whilst other biomarkers relating to the L-arginine-linked pathways remained unchanged.

This observation is strengthened by the fact that we observed the same pattern of changes in two independent, distinct study populations, both ascending from 1,130 to 4,559 m within 20 h. The first study comprised fifty healthy, non-acclimatized lowlanders, while the second study comprised 13 non-acclimatized lowlanders with a history of at least one episode of HAPE during a previous high altitude exposure. The major difference in L-arginine-related biomarkers between both groups was a significantly higher baseline ADMA concentration in the second, HAPE-susceptible, group. Interestingly enough, this group showed no significant further increase in ADMA during 67 h of high altitude exposure, while we measured a significant, incremental elevation of circulating ADMA during 44 h of high altitude exposure in the first group. Both groups showed similar trends of SDMA elevation with a peak at about 20 h after ascent, a delayed reduction in L-citrulline/L-arginine ratio beyond 20 h after ascent, and a rapid drop in L-ornithine/L-arginine ratio that reached a minimum at about 20–24 h after ascent.

ADMA and SDMA are important endogenous regulators of vascular endothelial NO formation. ADMA directly interferes with NO synthase catalytic activity and, when present at high concentrations, reduces NO formation in a manner that is reversible by excess L-arginine (Böger, 2006). SDMA impairs cellular L-arginine uptake and thereby has an indirect effect on intracellular substrate availability for L-arginine metabolic pathways (Hannemann and Böger, 2022). The ratio of L-citrulline over L-arginine, i.e., of product over substrate for NO synthase, is a surrogate indicator for total NO synthase activity. This ratio decreased over time in a manner paralleling the increases in ADMA and SDMA, thus suggesting that the observed changes in dimethylarginines resulted in impaired NO production.

Impaired endothelial NO-mediated function has previously been shown to be present the systemic circulation of individuals prone to HAPE (Berger et al., 2005). A similar reduction in pulmonary endothelial NO release might contribute to exaggerated hypoxic pulmonary vasoconstriction (Berger et al., 2009). This finding is also supported by an early experimental study (Archer et al., 1989). Measurements of exhaled NO in high altitude sojourners showed increases in pulmonary NO release in healthy individuals, but constant levels in HAPE-prone individuals (Duplain et al., 2000). At low altitude, Busch and co-workers found that exhaled NO was unchanged in normal individuals, but fell in HAPE-prone individuals (Busch et al., 2001). These data corroborate the validity of our biomarker findings in the present study.

L-ornithine is another metabolite derived from L-arginine by the enzymatic activity of arginases. Thus, the L-ornithine/L-arginine ratio serves as a surrogate of arginase activity. Our results therefore suggest a rapid and short-lived decline of arginase activity in non-HAPE-susceptible individuals, and a more prolonged decline of arginase activity in HAPE-susceptible individuals. As arginase competes with NO synthase for binding of L-arginine as a substrate, diminished arginase activity may be a counter-regulatory mechanism aimed at sparing intracellular L-arginine (Durante et al., 2007; Didelija et al., 2017). However, previous experimental and clinical investigations during more prolonged sojourns of humans or animals at high altitude reported increased arginase activity (Jiang et al., 2015; Lüneburg et al., 2016). Whether increased arginase activity contributes to, or whether diminished arginase activity ameliorates hypoxic pulmonary vasoconstriction remains to be settled. Further, our metabolite ratios offer a global view on whole-body enzymatic activity and may be influenced by other metabolic pathways involving L-arginine and/or L-ornithine. Notably, for example, the changes in SDMA that we observed in the two study cohorts may be caused by increased SDMA synthesis due to hypoxic upregulation of protein arginine N-methyltransferases, which we observed in a previous experimental study using lung tissue from mice exposed to 21 days of hypoxia (Hannemann et al., 2020a), or, alternatively, by diminished SDMA metabolism by its metabolizing enzyme, alanine glyoxylate aminotransferase-2 (AGXT2). AGXT2 is the only known enzyme that metabolizes SDMA (Lüneburg et al., 2014); however, its expression is described almost exclusively in the liver and kidneys (Jarzebska et al., 2019), which would point to a systemic and not purely local pulmonary effect of high altitude hypoxia.

In previous studies, we have observed elevations of ADMA during long-term hypoxia in rodent models (Lüneburg et al., 2016; Hannemann et al., 2020b) and during chronic-intermittent hypoxia during follow-up periods of up to 6 months (Lüneburg et al., 2017; Siques et al., 2019). The results of our present study extend these observations by showing that much shorter time periods of high altitude sojourn are sufficient to induce the same biochemical changes.

Enhanced hypoxic pulmonary vasoconstriction may cause a relevant pathological sequela, especially when maintained for prolonged periods of time. HAPH is a well-described long-term consequence of chronic and chronic-intermittent hypoxia (Brito et al., 2018; Siques et al., 2019). For the diagnosis of HAPH, the threshold of mean pulmonary arterial pressure had been set at 30 mmHg rather than the former 25 mmHg cut-off (most recently now 20 mmHg (Humbert et al., 2022) used to define pulmonary hypertension in lowlanders, in order to account for the fact that most individuals develop some level of mildly elevated pulmonary arterial pressure at altitude (León-Velarde et al., 2005). In the present study, SDMA, but not ADMA was positively correlated with RVPG, and L-ornithine and the L-ornithine/L-arginine ratio were negatively correlated with RVPG. These observations fit with the pathophysiological links between the biomarkers and pulmonary vascular tone as discussed above, as well as to the rapid onset of changes in the plasma concentrations of SDMA and L-ornithine after ascent to high altitude, which contrasted with the more delayed elevation of ADMA.

HAPE is an acute complication occurring at high altitude in non-acclimatized individuals and has been demonstrated to be a complication of acute excessive elevations of pulmonary arterial pressure at high altitude (Dehnert et al., 2007; Swenson and Bärtsch, 2012). HAPE-susceptible individuals have an abnormally sensitive pulmonary arteriolar response even to short challenges of hypoxia (Hultgren et al., 1971) or even normoxic exercise (Eldridge et al., 1996; Grünig et al., 2000; Swenson and Bärtsch, 2012), both of which have also been linked to reduced NO bioavailability (Berger et al., 2005). Treatment with drugs that cause pulmonary vasodilation such as nifedipine and tadalafil lower the incidence of HAPE by more than 80% (Bärtsch et al., 1991; Maggiorini et al., 2006). Experimental data also support the notion that fluid leakiness of the pulmonary endothelium is enhanced by low NO bioavailability in hypoxia (Kolluru et al., 2008). We found an association of ADMA concentrations with the incidence of HAPE in study B, which supports these pathophysiological concepts.

As in previous studies with more prolonged high altitude exposure, we observed no clinically meaningful associations of ADMA with AMS; however, a reproducible negative correlation of L-ornithine levels with AMS was observed. This may suggest involvement of elevated arginase activity in AMS pathophysiology, although due to the small sample size a type 2 error cannot be excluded.

We have previously analysed the associations of single nucleotide polymorphisms in genes encoding for major enzymes involved in the L-arginine/dimethylarginine/NO pathway with the circulating concentrations of L-arginine-related biomarkers. The major gene linked to ADMA plasma concentration is DDAH1, i.e., the gene encoding for one of two variants of the enzyme that converts ADMA to L-citrulline (Leiper et al., 1999; Hannemann et al., 2022). SNPs in the DDAH2 gene are inversely associated with ADMA concentrations (Hannemann et al., 2022). By contrast, SNPs in the PRMT genes, which encode for a family of protein L-arginine methyltransferases that are involved in the formation of symmetrically or asymmetrically dimethylated L-arginine residues within proteins, have no associations with ADMA or SDMA concentrations (Hannemann et al., 2022). In the context of the present study, it is interesting to note that the minor allele of the DDAH1 gene and the major allele of the DDAH2 gene also confer genetic sensibility for HAPH, and that SNPs in the NOS3 and ARG2 genes also show associations with HAPH (Hannemann et al., 2021). It might be interesting to study whether the allele frequencies for these genes are also different in HAPE-susceptible individuals *versus* those who are not.

One strength of our study is that followed strict and standardized protocols that were kept as identical as possible between both studies. This included the ascent profile, the assessment of clinical data, the echocardiographic measurements that were made by the same experienced echo cardiographer, and the biochemical analyses of L-arginine-related metabolites by UPLC-MS/MS. Nonetheless, we cannot exclude minor differences in handling of samples at high altitude. In addition, based on the retrospective analysis of the studies, we were unable to correct RVPG measurements for cardiac output due to unavailability of cardiac output measurements to refine definition of HAPH. Also, whilst we carefully checked our data for absence of any pharmacological effects of the drug treatments tested in each of the two study populations, we cannot formally exclude that a possible pharmacological effect on pulmonary hemodynamics that may have remained undetected could have affected the relation of RVPG to L-arginine metabolite concentrations.

In conclusion, we report here that ADMA and SDMA, two biomarkers influencing vascular endothelial NO production, are elevated during short exposure to high altitude in non-acclimatized individuals. Baseline ADMA concentration is high in HAPE-prone individuals, suggesting a possible involvement of these biomarkers not only in the long-term consequences of chronic or chronic-intermittent exposure to hypobaric hypoxia, but also an association with acute high altitude diseases like HAPE. If this hypothesis is correct, one would expect oral supplementation with L-arginine to contribute to prevention of this acute high altitude illness. The observations that exhaled NO is reduced in HAPE-prone individuals during high altitude exposure, and that administration of tadalafil, which prevents the breakdown of cyclic GMP by phosphodiesterase V, helps in the prevention of HAPE and by lowering pulmonary arterial pressure, support this hypothesis. Studies with L-arginine supplementation in hypobaric hypoxia are ongoing.

## Data Availability

The original contributions presented in the study are included in the article/Supplementary material, further inquiries can be directed to the corresponding author.

## References

[B1] Archer S. L. Tolins J. P. Raij L. Weir E. K. (1989). Hypoxic pulmonary vasoconstriction is enhanced by inhibition of the synthesis of an endothelium derived relaxing factor. Biochem. Biophys. Res. Commun. 164 (3), 1198–1205. 10.1016/0006-291x(89)91796-8 2480112

[B2] Bärtsch P. Bailey D. M. Berger M. M. Knauth M. Baumgartner R. W. (2004). Acute mountain sickness: controversies and advances. High. Alt. Med. Biol. 5 (2), 110–124. 10.1089/1527029041352108 15265333

[B3] Bärtsch P. Maggiorini M. Ritter M. Noti C. Vock P. Oelz O. (1991). Prevention of high-altitude pulmonary edema by nifedipine. N. Engl. J. Med. 325 (18), 1284–1289. 10.1056/NEJM199110313251805 1922223

[B4] Bärtsch P. Swenson E. R. (2013). Clinical practice: acute high-altitude illnesses. N. Engl. J. Med. 368 (24), 2294–2302. 10.1056/NEJMcp1214870 23758234

[B5] Beidleman B. A. Muza S. R. Fulco C. S. Rock P. B. Cymerman A. (2007). Validation of a shortened electronic version of the environmental symptoms questionnaire. High. Alt. Med. Biol. 8 (3), 192–199. 10.1089/ham.2007.1016 17824819

[B6] Berger M. M. Dehnert C. Bailey D. M. Luks A. M. Menold E. Castell C. (2009). Transpulmonary plasma ET-1 and nitrite differences in high altitude pulmonary hypertension. High. Alt. Med. Biol. 10 (1), 17–24. 10.1089/ham.2008.1053 19326597

[B7] Berger M. M. Hesse C. Dehnert C. Siedler H. Kleinbongard P. Bardenheuer H. J. (2005). Hypoxia impairs systemic endothelial function in individuals prone to high-altitude pulmonary edema. Am. J. Respir. Crit. Care Med. 172 (6), 763–767. 10.1164/rccm.200504-654OC 15947284

[B8] Berger M. M. Macholz F. Sareban M. Schmidt P. Fried S. Dankl D. (2017). Inhaled budesonide does not prevent acute mountain sickness after rapid ascent to 4559 m. Eur. Respir. J. 50 (3), 1700982. 10.1183/13993003.00982-2017 28890439

[B9] Berger M. M. Macholz F. Schmidt P. Fried S. Perz T. Dankl D. (2018). Inhaled budesonide does not affect hypoxic pulmonary vasoconstriction at 4559 meters of altitude. High. Alt. Med. Biol. 19 (1), 52–59. 10.1089/ham.2017.0113 29298124

[B10] Berger M. M. Sareban M. Schiefer L. M. Swenson K. E. Treff F. Schäfer L. (2022). Effects of acetazolamide on pulmonary artery pressure and prevention of high-altitude pulmonary edema after rapid active ascent to 4,559 m. J. Appl. Physiol. 132 (6), 1361–1369. 10.1152/japplphysiol.00806.2021 35511718

[B11] Böger R. Hannemann J. (2020). Dual role of the L-arginine-ADMA-NO pathway in systemic hypoxic vasodilation and pulmonary hypoxic vasoconstriction. Pulm. Circ. 10 (2), 2045894020918850. 10.1177/2045894020918850 32313645 PMC7153195

[B12] Böger R. H. (2006). Asymmetric dimethylarginine (ADMA): a novel risk marker in cardiovascular medicine and beyond. Ann. Med. 38 (2), 126–136. 10.1080/07853890500472151 16581698

[B13] Brito J. Siques P. López R. Romero R. León-Velarde F. Flores K. (2018). Long-term intermittent work at high altitude: right heart functional and morphological status and associated cardiometabolic factors. Front. Physiol. 9, 248. 10.3389/fphys.2018.00248 29623044 PMC5874329

[B14] Busch T. Bärtsch P. Pappert D. Grünig E. Hildebrandt W. Elser H. (2001). Hypoxia decreases exhaled nitric oxide in mountaineers susceptible to high-altitude pulmonary edema. Am. J. Respir. Crit. Care Med. 163 (2), 368–373. 10.1164/ajrccm.163.2.2001134 11179108

[B15] Dehnert C. Berger M. M. Mairbäurl H. Bärtsch P. (2007). High altitude pulmonary edema: a pressure-induced leak. Respir. Physiol. Neurobiol. 158 (2-3), 266–273. 10.1016/j.resp.2007.05.002 17602898

[B16] Didelija I. C. Mohammad M. A. Marini J. C. (2017). Ablation of arginase II spares arginine and abolishes the arginine requirement for growth in male mice. J. Nutr. 147 (8), 1510–1516. 10.3945/jn.117.251249 28679627 PMC5525112

[B17] Duplain H. Sartori C. Lepori M. Egli M. Allemann Y. Nicod P. (2000). Exhaled nitric oxide in high-altitude pulmonary edema: role in the regulation of pulmonary vascular tone and evidence for a role against inflammation. Am. J. Respir. Crit. Care Med. 162 (1), 221–224. 10.1164/ajrccm.162.1.9908039 10903245

[B18] Durante W. Johnson F. K. Johnson R. A. (2007). Arginase: a critical regulator of nitric oxide synthesis and vascular function. Clin. Exp. Pharmacol. Physiol. 34 (9), 906–911. 10.1111/j.1440-1681.2007.04638.x 17645639 PMC1955221

[B19] Eldridge M. W. Podolsky A. Richardson R. S. Johnson D. H. Knight D. R. Johnson E. C. (1996). Pulmonary hemodynamic response to exercise in subjects with prior high-altitude pulmonary edema. J. Appl. Physiol. 81 (2), 911–921. 10.1152/jappl.1996.81.2.911 8872663

[B20] Fenn W. O. Rahn H. Otis A. B. (1946). A theoretical study of the composition of the alveolar air at altitude. Am. J. Physiol. 146, 637–653. 10.1152/ajplegacy.1946.146.5.637 20996488

[B21] Ghofrani H. A. Voswinckel R. Reichenberger F. Weissmann N. Schermuly R. T. Seeger W. (2006). Hypoxia- and non-hypoxia-related pulmonary hypertension - established and new therapies. Cardiovasc Res. 72 (1), 30–40. 10.1016/j.cardiores.2006.07.025 16934242

[B22] Grünig E. Mereles D. Hildebrandt W. Swenson E. R. Kübler W. Kuecherer H. (2000). Stress Doppler echocardiography for identification of susceptibility to high altitude pulmonary edema. J. Am. Coll. Cardiol. 35 (4), 980–987. 10.1016/s0735-1097(99)00633-6 10732898

[B23] Hannemann J. Böger R. (2022). Dysregulation of the nitric oxide/dimethylarginine pathway in hypoxic pulmonary vasoconstriction-molecular mechanisms and clinical significance. Front. Med. (Lausanne) 9, 835481. 10.3389/fmed.2022.835481 35252268 PMC8891573

[B24] Hannemann J. Glatzel A. Hillig J. Zummack J. Schumacher U. Lüneburg N. (2020a). Upregulation of DDAH2 limits pulmonary hypertension and right ventricular hypertrophy during chronic hypoxia in Ddah1 knockout mice. Front. Physiol. 11, 597559. 10.3389/fphys.2020.597559 33281630 PMC7689360

[B25] Hannemann J. Siques P. Schmidt-Hutten L. Zummack J. Brito J. Böger R. (2021). Association of genes of the NO pathway with altitude disease and hypoxic pulmonary hypertension. J. Clin. Med. 10 (24), 5761. 10.3390/jcm10245761 34945057 PMC8704804

[B26] Hannemann J. Zummack J. Hillig J. Böger R. (2020b). Metabolism of asymmetric dimethylarginine in hypoxia: from bench to bedside. Pulm. Circ. 10 (2), 2045894020918846. 10.1177/2045894020918846 32313644 PMC7158260

[B27] Hannemann J. Zummack J. Hillig J. Rendant-Gantzberg L. Böger R. (2022). Association of variability in the DDAH1, DDAH2, AGXT2 and PRMT1 genes with circulating ADMA concentration in human whole blood. J. Clin. Med. 11 (4), 941. 10.3390/jcm11040941 35207213 PMC8877358

[B28] Hultgren H. N. Grover R. F. Hartley L. H. (1971). Abnormal circulatory responses to high altitude in subjects with a previous history of high-altitude pulmonary edema. Circulation 44 (5), 759–770. 10.1161/01.cir.44.5.759 5115068

[B29] Humbert M. Kovacs G. Hoeper M. M. Badagliacca R. Berger R. M. F. Brida M. (2022). 2022 ESC/ERS Guidelines for the diagnosis and treatment of pulmonary hypertension. Eur. Heart J. 43 (38), 3618–3731. 10.1093/eurheartj/ehac237 36017548

[B30] Jarzebska N. Georgi S. Jabs N. Brilloff S. Maas R. Rodionov R. N. (2019). Kidney and liver are the main organs of expression of a key metabolic enzyme alanine:glyoxylate aminotransferase 2 in humans. Atheroscler. Suppl. 40, 106–112. 10.1016/j.atherosclerosissup.2019.08.041 31818439

[B31] Jiang W. Sun B. Song X. Zheng Y. Wang L. Wang T. (2015). Arginase inhibition protects against hypoxia-induced pulmonary arterial hypertension. Mol. Med. Rep. 12 (3), 4743–4749. 10.3892/mmr.2015.3994 26126810

[B32] Kolluru G. K. Tamilarasan K. P. Rajkumar A. S. Geetha Priya S. Rajaram M. Saleem N. K. (2008). Nitric oxide/cGMP protects endothelial cells from hypoxia-mediated leakiness. Eur. J. Cell Biol. 87 (3), 147–161. 10.1016/j.ejcb.2007.10.001 18023499

[B33] Krause B. J. Del Rio R. Moya E. A. Marquez-Gutierrez M. Casanello P. Iturriaga R. (2015). Arginase-endothelial nitric oxide synthase imbalance contributes to endothelial dysfunction during chronic intermittent hypoxia. J. Hypertens. 33 (3), 515–524. ; discussion 524. 10.1097/HJH.0000000000000453 25629363

[B34] Leiper J. M. Santa Maria J. Chubb A. MacAllister R. J. Charles I. G. Whitley G. S. (1999). Identification of two human dimethylarginine dimethylaminohydrolases with distinct tissue distributions and homology with microbial arginine deiminases. Biochem. J. 343 (1), 209–214. 10.1042/bj3430209 10493931 PMC1220543

[B35] León-Velarde F. Maggiorini M. Reeves J. T. Aldashev A. Asmus I. Bernardi L. (2005). Consensus statement on chronic and subacute high altitude diseases. High. Alt. Med. Biol. 6 (2), 147–157. 10.1089/ham.2005.6.147 16060849

[B36] López V. Uribe E. Moraga F. A. (2021). Activation of arginase II by asymmetric dimethylarginine and homocysteine in hypertensive rats induced by hypoxia: a new model of nitric oxide synthesis regulation in hypertensive processes? Hypertens. Res. 44 (3), 263–275. 10.1038/s41440-020-00574-1 33149269

[B37] Luks A. M. Hackett P. H. (2022). Medical conditions and high-altitude travel. N. Engl. J. Med. 386 (4), 364–373. 10.1056/NEJMra2104829 35081281

[B38] Lüneburg N. Lieb W. Zeller T. Chen M. H. Maas R. Carter A. M. (2014). Genome-wide association study of L-arginine and dimethylarginines reveals novel metabolic pathway for symmetric dimethylarginine. Circ. Cardiovasc Genet. 7 (6), 864–872. 10.1161/CIRCGENETICS.113.000264 25245031 PMC4797637

[B39] Lüneburg N. Siques P. Brito J. Arriaza K. Pena E. Klose H. (2016). Long-term chronic intermittent hypobaric hypoxia in rats causes an imbalance in the asymmetric dimethylarginine/nitric oxide pathway and ros activity: a possible synergistic mechanism for altitude pulmonary hypertension? Pulm. Med. 2016, 6578578. 10.1155/2016/6578578 27313889 PMC4904121

[B40] Lüneburg N. Siques P. Brito J. De La Cruz J. J. León-Velarde F. Hannemann J. (2017). Long-term intermittent exposure to high altitude elevates asymmetric dimethylarginine in first exposed young adults. High. Alt. Med. Biol. 18 (3), 226–233. 10.1089/ham.2016.0123 28453332 PMC5649417

[B41] Lüneburg N. Xanthakis V. Schwedhelm E. Sullivan L. M. Maas R. Anderssohn M. (2011). Reference intervals for plasma L-arginine and the L-arginine:asymmetric dimethylarginine ratio in the Framingham Offspring Cohort. J. Nutr. 141 (12), 2186–2190. 10.3945/jn.111.148197 22031661 PMC3223876

[B42] Maggiorini M. Brunner-La Rocca H. P. Peth S. Fischler M. Böhm T. Bernheim A. (2006). Both tadalafil and dexamethasone may reduce the incidence of high-altitude pulmonary edema: a randomized trial. Ann. Intern Med. 145 (7), 497–506. 10.7326/0003-4819-145-7-200610030-00007 17015867

[B43] Roach R. C. (1993). The Lake Louise acute mountain sickness scoring system. Hypoxia Mol. Med., 272–274.

[B44] Roach R. C. Hackett P. H. Oelz O. Bärtsch P. Luks A. M. MacInnis M. J. (2018). The 2018 Lake Louise acute mountain sickness score. High. Alt. Med. Biol. 19 (1), 4–6. 10.1089/ham.2017.0164 29583031 PMC6191821

[B45] Rossetti G. M. K. Macdonald J. H. Wylie L. J. Little S. J. Newton V. Wood B. (2017). Dietary nitrate supplementation increases acute mountain sickness severity and sense of effort during hypoxic exercise. J. Appl. Physiol. 123 (4), 983–992. 10.1152/japplphysiol.00293.2017 28684588

[B46] Scherrer U. Vollenweider L. Delabays A. Savcic M. Eichenberger U. Kleger G. R. (1996). Inhaled nitric oxide for high-altitude pulmonary edema. N. Engl. J. Med. 334 (10), 624–629. 10.1056/NEJM199603073341003 8592525

[B47] Schwedhelm E. Maas R. Tan-Andresen J. Schulze F. Riederer U. Böger R. H. (2007). High-throughput liquid chromatographic-tandem mass spectrometric determination of arginine and dimethylated arginine derivatives in human and mouse plasma. J. Chromatogr. B Anal. Technol. Biomed. Life Sci. 851 (1-2), 211–219. 10.1016/j.jchromb.2006.11.052 17194630

[B48] Schwedhelm E. Xanthakis V. Maas R. Sullivan L. M. Atzler D. Lüneburg N. (2011). Plasma symmetric dimethylarginine reference limits from the Framingham offspring cohort. Clin. Chem. Lab. Med. 49 (11), 1907–1910. 10.1515/CCLM.2011.679 21864208 PMC3235736

[B49] Schwedhelm E. Xanthakis V. Maas R. Sullivan L. M. Schulze F. Riederer U. (2009). Asymmetric dimethylarginine reference intervals determined with liquid chromatography-tandem mass spectrometry: results from the Framingham offspring cohort. Clin. Chem. 55 (8), 1539–1545. 10.1373/clinchem.2009.124263 19541865 PMC3794429

[B50] Siques P. Brito J. Schwedhelm E. Pena E. León-Velarde F. De La Cruz J. J. (2019). Asymmetric dimethylarginine at sea level is a predictive marker of hypoxic pulmonary arterial hypertension at high altitude. Front. Physiol. 10, 651. 10.3389/fphys.2019.00651 31191349 PMC6545974

[B51] Swenson E. R. (2013). Hypoxic pulmonary vasoconstriction. High. Alt. Med. Biol. 14 (2), 101–110. 10.1089/ham.2013.1010 23795729

[B52] Swenson E. R. Bärtsch P. (2012). High-altitude pulmonary edema. Compr. Physiol. 2 (4), 2753–2773. 10.1002/cphy.c100029 23720264

[B53] West J. B. (2012). High-altitude medicine. Am. J. Respir. Crit. Care Med. 186 (12), 1229–1237. 10.1164/rccm.201207-1323CI 23103737

[B54] West J. B. Hackett P. H. Maret K. H. Milledge J. S. Peters R. M. Jr. Pizzo C. J. (1983). Pulmonary gas exchange on the summit of Mount Everest. J. Appl. Physiol. Respir. Environ. Exerc Physiol. 55 (3), 678–687. 10.1152/jappl.1983.55.3.678 6415007

